# A theory of organizational readiness for change

**DOI:** 10.1186/1748-5908-4-67

**Published:** 2009-10-19

**Authors:** Bryan J Weiner

**Affiliations:** 1 Department of Health Policy and Management, Gillings School of Global Public Health, University of North Carolina Chapel Hill, Chapel Hill, North Carolina, USA

## Abstract

**Background:**

Change management experts have emphasized the importance of establishing organizational readiness for change and recommended various strategies for creating it. Although the advice seems reasonable, the scientific basis for it is limited. Unlike individual readiness for change, organizational readiness for change has not been subject to extensive theoretical development or empirical study. In this article, I conceptually define organizational readiness for change and develop a theory of its determinants and outcomes. I focus on the organizational level of analysis because many promising approaches to improving healthcare delivery entail collective behavior change in the form of systems redesign--that is, multiple, simultaneous changes in staffing, work flow, decision making, communication, and reward systems.

**Discussion:**

Organizational readiness for change is a multi-level, multi-faceted construct. As an organization-level construct, readiness for change refers to organizational members' shared resolve to implement a change (change commitment) and shared belief in their collective capability to do so (change efficacy). Organizational readiness for change varies as a function of how much organizational members value the change and how favorably they appraise three key determinants of implementation capability: task demands, resource availability, and situational factors. When organizational readiness for change is high, organizational members are more likely to initiate change, exert greater effort, exhibit greater persistence, and display more cooperative behavior. The result is more effective implementation.

**Summary:**

The theory described in this article treats organizational readiness as a shared psychological state in which organizational members feel committed to implementing an organizational change and confident in their collective abilities to do so. This way of thinking about organizational readiness is best suited for examining organizational changes where collective behavior change is necessary in order to effectively implement the change and, in some instances, for the change to produce anticipated benefits. Testing the theory would require further measurement development and careful sampling decisions. The theory offers a means of reconciling the structural and psychological views of organizational readiness found in the literature. Further, the theory suggests the possibility that the strategies that change management experts recommend are equifinal. That is, there is no 'one best way' to increase organizational readiness for change.

## Background

Organizational readiness for change is considered a critical precursor to the successful implementation of complex changes in healthcare settings [1-9]. Indeed, some suggest that failure to establish sufficient readiness accounts for one-half of all unsuccessful, large-scale organizational change efforts [6]. Drawing on Lewin's [10] three-stage model of change, change management experts have prescribed various strategies to create readiness by 'unfreezing' existing mindsets and creating motivation for change. These strategies include highlighting the discrepancy between current and desired performance levels, fomenting dissatisfaction with the status quo, creating an appealing vision of a future state of affairs, and fostering confidence that this future state can be achieved [2,4,11-16].

While this advice seems reasonable and useful, the scientific basis for these recommendations is limited. Unlike individual readiness for change, organizational readiness for change has not been subject to extensive empirical study [17]. Unfortunately, simply calling for more research will not do. As two recently published reviews indicate, most publicly available instruments for measuring organizational readiness for change exhibit limited evidence of reliability or validity [17,18]. At a more basic level, these reviews reveal conceptual ambiguity about the meaning of organizational readiness for change and little theoretically grounded discussion of the determinants or outcomes of organizational readiness. In the absence of theoretical clarification and exploration of these issues, efforts to advance measurement, produce cumulative knowledge, and inform practice will likely remain stalled.

In this article, I conceptually define organizational readiness for change and develop a theory of its determinants and outcomes. Although readiness is a multi-level construct, I focus on the supra-individual levels of analysis because many promising approaches to improving healthcare delivery entail collective behavior change in the form of systems redesign--that is, multiple, simultaneous changes in staffing, work flow, decision making, communication, and reward systems. In exploring the meaning of organizational readiness and offering a theory of its determinants and outcomes, my intent is to promote further scholarly discussion and stimulate empirical inquiry of an important, yet under-studied topic in implementation science.

## Discussion

### What is organizational readiness for change?

Organizational readiness for change is a multi-level construct. Readiness can be more or less present at the individual, group, unit, department, or organizational level. Readiness can be theorized, assessed, and studied at any of these levels of analysis. However, organizational readiness for change is not a homologous multi-level construct [19]. That is, the construct's meaning, measurement, and relationships with other variables differ across levels of analysis [17,20]. Below, I focus on organizational readiness for change as a supra-individual state of affairs and theorize about its organizational determinants and organizational outcomes.

Organizational readiness for change is not only a multi-level construct, but a multi-faceted one. Specifically, organizational readiness refers to organizational members' change commitment and change efficacy to implement organizational change [17,20]. This definition followed the ordinary language use of the term 'readiness,' which connotes a state of being both psychologically and behaviorally prepared to take action (*i.e.*, willing and able). Similar to Bandura's [21] notion of goal commitment, change commitment to change refers to organizational members' shared resolve to pursue the courses of action involved in change implementation. I emphasize shared resolve because implementing complex organizational changes involves collective action by many people, each of whom contributes something to the implementation effort. Because implementation is often a 'team sport,' problems arise when some feel committed to implementation but others do not. Herscovitch and Meyer [22] observe that organizational members can commit to implementing an organizational change because they want to (they value the change), because they have to (they have little choice), or because they ought to (they feel obliged). Commitment based on 'want to' motives reflects the highest level of commitment to implement organizational change.

Like Bandura's [21] notion of collective efficacy, change efficacy refers to organizational members' shared beliefs in their collective capabilities to organize and execute the courses of action involved in change implementation. Here again, I emphasize shared beliefs and collective capabilities because implementation entails collective (or conjoint) action among interdependent individuals and work units. Coordinating action across many individuals and groups and promoting organizational learning are good examples of collective (or conjoint) capabilities. As Bandura and others note, efficacy judgments refer to action capabilities; efficacy judgments are neither outcome expectancies [23-25] nor assessments of knowledge, skills, or resources [23]. Change efficacy is higher when people share a sense of confidence that collectively they can implement a complex organizational change.

Several points about this conceptual definition of organizational readiness for change merit discussion. First, organizational readiness for change is conceived here in psychological terms. Others describe organizational readiness for change in more structural terms, emphasizing the organization's financial, material, human, and informational resources [26-34].

In the theory presented here, organizational structures and resource endowments shape readiness perceptions. In other words, organizational members take into consideration the organization's structural assets and deficits in formulating their change efficacy judgments. Second, organizational readiness for change is situational; it is not a general state of affairs. Some organizational features do seem to create a more receptive context for innovation and change [35-37]. However, receptive context does not translate directly into readiness. The content of change matters as much as the context of change. A healthcare organization could, for example, exhibit a culture that values risk-taking and experimentation a positive working environment (*e.g.*, good managerial-clinical relationships), and a history of successful change implementation. Yet, despite this receptive context, this organization could still exhibit a high readiness to implement electronic medical records, but a low readiness to implement an open-access scheduling system. Commitment is, in part, change specific; so too are efficacy judgments. It is possible that receptive context is a necessary but not sufficient condition for readiness. For example, good managerial-clinical relationships might be necessary for promoting any change even if it does not guarantee that clinicians will commit to implementing a specific change. The theory proposed here embraces this possibility by regarding receptive organizational context features as possible determinants of readiness rather than readiness itself. Third, the two facets of organizational readiness for change--change commitment and change efficacy--are conceptually interrelated and, I expect, empirically correlated. As Bandura [21] notes, low levels of confidence in one's capabilities to execute a course of action can impair one's motivation to engage in that course of action. Likewise, as Maddux [25] notes, fear and other negative motivational states can lead one to underestimate or downplay one's judgments of capability. These cognitive and motivational aspects of readiness are expected to covary, but not to covary perfectly. At one extreme, organizational members could be very confident that they could implement an organizational change successfully, yet show little or no motivation to do so. The opposite extreme is also possible, as are all points in between. Organizational readiness is likely to be highest when organizational members not only want to implement an organizational change and but also feel confident that they can do so.

What circumstances are likely to generate a shared sense of readiness? Consistent leadership messages and actions, information sharing through social interaction, and shared experience--including experience with past change efforts--could promote commonality in organizational members' readiness perceptions [19]. Broader organizational processes like attraction, selection, socialization, and attrition might also play a role [38-40]. Conversely, organizational members are unlikely to hold common perceptions of readiness when leaders communicate inconsistent messages or act in inconsistent ways, when intra-organizational groups or units have limited opportunity to interact and share information, or when organizational members do not have a common basis of experience. Intra-organizational variability in readiness perceptions indicates lower organizational readiness for change and could signal problems in implementation efforts that demand coordinated action among interdependent actors.

### What conditions promote organizational readiness for change?

**Figure 1:**
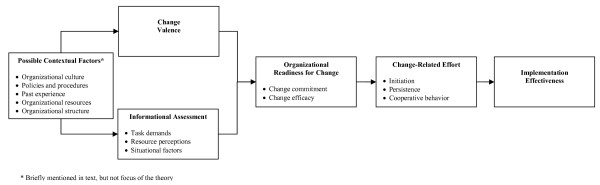
**Determinants and Outcomes of Organizational Readiness for Change**. Included in separate document, per instructions to authors concerning figures.

If generating a shared sense of readiness sounds difficult, that is because it probably is. This might explain why many organizations fail to generate sufficient organizational readiness and, consequently, experience problems or outright failure when implementing complex organizational change. Although organizational readiness for change is difficult to generate, motivation theory and social cognitive theory suggest several conditions or circumstances that might promote it (see Figure 1).

### Change valence

Drawing on motivation theory [41-43], I propose that change commitment is largely a function of change valence. Simply put, do organizational members value the specific impending change? For example, do they think that it is needed, important, beneficial, or worthwhile? The more organizational members value the change, the more they will want to implement the change, or, put differently, the more resolve they will feel to engage in the courses of action involved in change implementation. Change valence is a parsimonious construct that brings some theoretical coherence to the numerous and disparate drivers of readiness that change management experts and scholars have discussed [11,13,22,28,44-46]. Organizational members might value a planned organizational change because they believe some sort of change is urgently needed. They might value it because they believe the change is effective and will solve an important organizational problem. They might value it because they value the benefits that they anticipate the organizational change will produce for the organization, patients, employees, or them personally. They might value it because it resonates with their core values. They might value it because managers support it, opinion leaders support it, or peers support it. Given the many reasons why organizational members might value an organizational change, it seems unlikely that any of these specific reasons will exhibit consistent, cross-situational relationships with organizational readiness for change. In fact, it might not be necessary that all organizational members value an organizational change for the same reasons. Change valence resulting from disparate reasons might be just as potent a determinant of change commitment as change valence resulting from commonly shared reasons. For organizational readiness, the key question is: regardless of their individual reasons, do organizational members collectively value the change enough to commit to its implementation?

### Change efficacy

Drawing on social cognitive theory, and specifically the work of Gist and Mitchell [47], I propose that change efficacy is a function of organizational members' cognitive appraisal of three determinants of implementation capability: task demands, resource availability, and situational factors. As Gist and Michell [[47]:184] observe, efficacy is a 'comprehensive summary or judgment of perceived capability to perform a task.' In formulating change-efficacy judgments, organizational members acquire, share, assimilate, and integrate information bearing on three questions: do we know what it will take to implement this change effectively; do we have the resources to implement this change effectively; and can we implement this change effectively given the situation we currently face? Implementation capability depends in part on knowing what courses of action are necessary, what kinds of resources are needed, how much time is needed, and how activities should be sequenced. In addition to gauging knowledge of task demands, organizational members also cognitively appraise the match between task demands and available resources. That is, they assess whether the organization has the human, financial, material, and informational resources necessary to implement the change well. Finally, they consider situational factors such as, for example, whether sufficient time exists to implement the change well or whether the internal political environment supports implementation. When organizational members share a common, favorable assessment of task demands, resource availability, and situational factors, they share a sense of confidence that collectively they can implement a complex organizational change. In other words, change efficacy is high.

### Contextual factors

Change management experts and scholars have discussed other, broader contextual conditions that affect organizational readiness for change. For example, some contend that an organizational culture that embraces innovation, risk-taking, and learning supports organizational readiness for change [48-51]. Others stress the importance of flexible organizational policies and procedures and positive organizational climate (*e.g.*, good working relationships) in promoting organizational readiness [52-54]. Still others suggest that positive past experience with change can foster organizational readiness [2]. I contend that these broader, contextual conditions affect organizational readiness through the more proximal conditions described above. Organizational culture, for example, could amplify or dampen the change valence associated with a specific organizational change, depending on whether the change effort fits or conflicts with cultural values. Likewise, organizational policies and procedures could positively or negatively affect organizational members' appraisals of task demands, resource availability, and situational factors. Finally, past experience with change could positive or negatively affect organizational members' change valence (*e.g.*, whether they think the change really will deliver touted benefits) and change efficacy judgments (*e.g.*, whether they think the organization can effectively execute and coordinate change-related activities).

### What outcomes result from organizational readiness for change?

Outcomes are perhaps the least theorized and least studied aspect of organizational readiness for change. Change experts assert that greater readiness leads to more successful change implementation. But how, or why, is this so? Social cognitive theory suggests that when organizational readiness for change is high, organizational members are more likely to initiate change (*e.g.*, institute new policies, procedures, or practices), exert greater effort in support of change, and exhibit greater persistence in the face of obstacles or setbacks during implementation [21,47]. Motivation theory not only supports these hypotheses, but suggests another [22,41-43]. When organizational readiness is high, organizational members will exhibit more pro-social, change-related behavior--that is, actions supporting the change effort that exceed job requirements or role expectations. Research by Herscovitch and Meyer [22] supports this contention. They found that organizational members whose commitment to change was based on (*i.e.*, determined by) 'want to' motives rather than 'need to' motives or 'ought to' motives exhibited not only more cooperative behavior (*e.g.*, volunteering for problem-solving teams), but also championing behavior (*e.g.*, promoting the value of the change to others).

What is the end result of all this change-related effort? Drawing on implementation theory, the most proximal outcome is likely to be effective implementation. Following Klein and Sorra [55], implementation effectiveness refers to the consistency and quality of organizational members' initial or early use of a new idea, program, process, practice, or technology. To illustrate, when organizational readiness for change is high, community health centers providers and staff will more skillfully and persistently take action to put a diabetes registry in practice and demonstrate more consistent, high-quality use of the registry. By contrast, when organizational readiness for change is low or nonexistent, community health center providers and staff will resist initiating change, put less effort into implementation, persevere less in the face of implementation challenges, and exhibit compliant registry use, at best. In the absence of further intervention, registry use is likely to be intermittent, scattered, and uneven.

Organizational readiness for change does not guarantee that the implementation of a complex organizational change will succeed in terms of improving quality, safety, efficiency or some other anticipated outcome. Implementation effectiveness is a necessary, but not sufficient condition for achieving positive outcomes [55]. If the complex organizational change is poorly designed, or if it lacks efficacy, no amount of consistent, high-quality use will generate anticipated benefits. Moreover, it is important to recognize that organizational members can misjudge organizational readiness by, for example, overestimating (or even underestimating) their collective capabilities to implement the change. As Bandura [21,23] notes, efficacy judgments based on rich, accurate information, preferably based on direct experience, are more predictive than those based on incomplete or erroneous information.

### Some thoughts on testing this theory

Because this theory of organizational readiness for change is pitched at the organizational level of analysis, a test of the theory's predictions would require a multi-organization research design in which a set of organizations implements a common, or at least comparable, complex organizational change. A large healthcare system implementing Six Sigma or lean manufacturing on a system-wide basis would provide a useful opportunity to test the theory. So too would an association of community health centers agreeing to implement a common multi-component diabetes management program, or a group of affiliated specialty practices deciding to implement a common electronic medical record.

Could the theory be tested at the clinic, department, or divisional level? The idea of testing the theory at an intra-organizational level of analysis holds some appeal given sample size and statistical power considerations. If a reasonable case can be made that the clinics, departments, or divisions are distinct units of implementation (*e.g.*, they have some autonomy in change implementation), then the idea of testing the theory at an intra-organizational level of analysis seems defensible. However, careful consideration should be given to the question of whether the construct's meaning, measurement, and functional relations change by moving to the analysis down to intra-organizational level.

It is important to note that organizational readiness for change is conceptualized here as a 'shared team property'--that is, a psychological state that organizational members hold in common [19]. The extent to which this shared psychological state exists in any given situation is an empirical issue requiring the examination of within-group agreement statistics. If sufficient within-group agreement exists (*i.e.*, organizational members agree in their readiness perceptions), then analysis of organizational readiness as a shared team property can proceed. If insufficient within-group agreement exists (*i.e.*, organizational members disagree in their readiness perceptions), organizational readiness as a shared team property does not exist. Instead, the analyst must either focus on a lower level of analysis (*e.g.*, team readiness) or conceptualize organizational readiness as a configural property and theorize about the determinants and outcomes of intra-organizational variability in readiness perceptions [19].

Finally, as noted earlier, most publicly available instruments for measuring organizational readiness for change exhibit limited evidence of reliability and validity. As two recently published reviews indicate, most of the instruments employed in peer-reviewed research were not developed systematically using theory, nor were they subjected to extensive psychometric testing [17,18]. There are a few instruments have undergone thorough psychometric assessment. However, none of these instruments is suitable for measuring organizational readiness for change as defined above, either because they focus on individual readiness rather than organizational readiness, or because they treat readiness as a general state of affairs rather than something change-specific, or because they include items that the theory presented above considers determinants of readiness rather than readiness itself (*e.g.*, items pertaining to change valence). Although it is beyond the scope of this article to discuss measurement issues in detail, an instrument that would best fit the construct of readiness as described above would have the following characteristics:

1. Some means of focusing respondents' attention on a specific impending organizational change, perhaps by including a brief description of the change in the survey instrument and by mentioning the change by name in the instructions for specific item sets.

2. Group-referenced rather than self-referenced items (*e.g.*, items focusing on collective commitment and capabilities rather than personal commitment and capabilities).

3. Items that only capture change commitment or change efficacy, not related constructs, like the antecedent conditions discussed above (Nunnally [56] refers to such items as direct measures).

4. Efficacy items that are tailored to the specific organizational change, yet not so tailored that that the instrument could be used in other circumstances without substantial modification.

Satisfying this last point would be challenging, but it does not seem impossible. Health behavior scientists have successfully developed self-efficacy instruments for smoking, physical activity, and other health behaviors that are reliable and valid within their domain of application [57-63]. Although item content is tailored, the instruments are based on theory and have enough features in common that scholars can accumulate scientific knowledge across health problems. With respect to organizational readiness for change, it might be possible to identify a set of frequently occurring courses of action that must be skillfully organized and executed to achieve effective implementation of complex organizational changes. Possible candidates include: developing an effective strategy or plan for implementing the change; getting people involved and invested in implementing the change; coordinating tasks so that implementation goes smoothly; anticipating or preventing problems that might arise during implementation; and managing the politics of implementing the change. A pool of items could perhaps be developed that researchers could use in order to construct organizational readiness for change instruments that fit specific change contexts, yet share at least some content with other tailored instruments.

## Summary

In this article, I sought to conceptually define organizational readiness for change and develop a theory of its determinants and outcomes. In contrast to much of the literature on the topic, the conceptual definition offered here treats organizational readiness as a shared team property--that is, a shared psychological state in which organizational members feel committed to implementing an organizational change and confident in their collective abilities to do so. This way of thinking about organizational readiness is best suited for organizational changes where collective, coordinated behavior change is necessary in order to effectively implement the change and, in some instances, for the change to produce anticipated benefits. Some of the most promising organizational changes in healthcare delivery require collective, coordinated behavior change by many organizational members. Electronic health records, chronic care models, open access scheduling, quality improvement programs, and patient safety systems are but a few examples. There are, however, many evidence-based practices that providers could adopt, implement, and use on their own with relatively modest training or support (*e.g.*, smoking cessation counseling, foot exams for diabetic patients). Often such practices can generate benefits for individual providers, or their patients, regardless of whether other providers also adopt, implement, or use them. Individual-level theories of behavior change--such as the theory of planned behavior or the trans-theoretical model of change--apply more readily to such cases than organization-level theories do because the adoption, implementation, use, and outcomes of such evidence-based practices do not depend on collective, coordinated behavior change. The greater the degree of interdependence in change processes and outcomes, the greater the utility of supra-individual theories of readiness, such as the one presented here.

The article makes three contributions to theory and research. First, the article's discussion of the meaning of organizational readiness addresses a fundamental conceptual ambiguity that runs through the literature on the topic: is readiness a structural construct or a psychological one? The theory that I describe seeks to reconcile the structural view and psychological view by specifying a relationship between them. In this theory, resources and other structural attributes of organizations do not enter directly into the definition of readiness. Instead, they represent an important class of performance determinants that organizational members consider in formulating change efficacy judgments. This view is consistent with Bandura's [21] contention that efficacy judgments focus on generative capabilities--that is, the capability to mobilize resources and orchestrate courses of action to produce a skillful performance. Thus, organizations with the same resources, endowments, and organizational structures can differ in the effectiveness with which they implement the same organizational change depending on how they utilize, combine, and sequence organizational resources and routines. It seems preferable to regard organizational structures and resource endowments as capacity to implement change rather than readiness to do so. This distinction between capacity and readiness could move theory and research forward by reducing some of the conceptual ambiguity in the meaning and use of the term 'readiness.'

Second, the article's discussion of determinants illuminates the theoretical basis for the various strategies that change management experts recommend for creating organizational readiness. For practitioners, it might not seem necessary to explain in theoretical terms how or why a strategy works. For researchers, however, theoretical explication of the pathways through which these strategies affect readiness is important for advancing scientific knowledge. The theory that I propose suggests that strategies such as highlighting the discrepancy between current and desired performance levels, fomenting dissatisfaction with the status quo, creating an appealing vision of a future state of affairs increase organizational readiness for change by increasing change valence--that is, by increasing the degree to which organizational members perceive the change as needed, important, or worthwhile. In addition to advancing scientific knowledge, identifying and testing the pathways through which actions (strategies) have effects can have practical implications as well. Such efforts can prompt the discovery of new strategies or alternative pathways, or they can show the equifinality of already known strategies. For example, in the theory that I describe, the keys to increasing readiness are raising change valence and promoting a positive assessment of task demands, resource availability, and situational factors. It seems unlikely that there is one best way to achieve these goals; at the same time, it seems unlikely that all ways are always equally effective. Creating a sense of urgency might be useful for increasing change valence in some situations (*i.e.*, when complacency is high), but not others (*i.e.*, when uncertainty is high). Likewise, end-user involvement in change design and implementation planning can be a powerful way for not only increasing change valence (*e.g.*, helping people to see why this change is needed, important, and worthwhile), but also for helping organizational members realistically appraise the match of task demands, available resources, and situational factors. When, for whatever reason, end-user involvement is not an appropriate or feasible strategy, vicarious learning strategies (*e.g.*, site visits) could be useful for supplying organizational members with accurate information about task demands, resource requirements, and situational factors affecting implementation. If readiness-enhancing strategies are indeed equifinal--and this is an empirical question--then organizational leaders, innovation champions, and other change agents could take with a grain of salt the 'one best way' advice so often found in prescriptive change management writing, and focus instead of developing and using strategies that are tailored to local needs, opportunities, and constraints.

Third, the article's discussion of outcomes develops a theoretical link between two disparate bodies of research: organizational readiness for change and implementation theory and research. As noted earlier, change experts have asserted that greater organizational readiness leads to more successful implementation without specifying what 'successful implementation' means or explaining how or why this might be so. This article uses implementation theory to conceptually define the notion of implementation effectiveness and distinguish implementation effectiveness from innovation effectiveness. Moreover, the article draws on social cognitive theory and motivation theory to explain how greater organizational readiness could result in more effective change implementation. Implementation theory could also benefit from a stronger theoretical link. Although it is beyond the scope of this article to discuss in detail, I suspect that the construct of implementation climate--which Klein and Sorra [55] define as organizational members' shared perception that innovation use is expected, supported, and rewarded--has much in common with organizational readiness for change, the principal difference being that one construct applies in the 'pre-implementation' period while the other applies once implementation has begun. This article merely begins the dialogue between these two bodies of research which hitherto have developed independently of one another. Whether or not the theory developed here ultimately finds empirical support, I hope that its discussion promotes scholarly debate and stimulates empirical inquiry into an important, yet under-studied topic in implementation science.

## Competing interests

The author declares that he has no competing interests.
